# Electron Microscopy for the Stability Assessment of Parenteral Nutrition Admixtures: Focus on Precipitation

**DOI:** 10.3390/nu16091390

**Published:** 2024-05-04

**Authors:** Luis Otero-Millán, Brais Bea-Mascato, Jose Luis Legido Soto, Noemi Martínez-López-De-Castro, Natividad Lago Rivero

**Affiliations:** 1 Pharmacy Department, University Hospital Complex of Vigo, 36312 Vigo, Spain; noemi.martinez.lopezdecastro@sergas.es; 2 NeumoVigo I+i Research Group, Galicia Sur Health Research Institute (IIS Galicia Sur) SERGAS-UVIGO, 36312 Vigo, Spain; 3 Innovation in Clinical Pharmacy Research Group (i-FARMA-Vigo), Galicia Sur Health Research Institute (IIS Galicia Sur) SERGAS-UVIGO, 36312 Vigo, Spain; brais.bea@iisgaliciasur.es; 4 Applied Physic Department, Faculty of Sciences, University of Vigo, 36310 Vigo, Spain; xllegido@uvigo.es

**Keywords:** precipitation, parenteral nutrition, stability

## Abstract

(1) Background: parenteral nutrition (PN) is indispensable for patients unable to receive oral or enteral feeding. However, the complexity of PN solutions presents challenges regarding stability and compatibility. Precipitation reactions may occur. The most frequent is the formation of calcium phosphate (Ca-P). The different factors influencing these reactions must be considered to ensure patient safety. (2) Methods: eight paediatric PN solutions were prepared, following standard protocols. Samples were stored at room temperature and in a refrigerator. Electron microscopy, coupled with energy dispersive X-ray spectroscopy (EDS), was employed. Precipitates were analysed for composition and morphology. (3) Results: precipitates were observed in all samples, even at day 0. Crystalline structures, predominantly composed of calcium or magnesium, sometimes associated with chlorine or phosphorus, were detected. Additionally, amorphous precipitates, contained heterogeneous compositions, including unexpected elements, were identified. (4) Conclusions: various precipitates, primarily calcium- or magnesium-based, can form in PN solutions, although it is not expected that they can form under the real conditions of use. Calcium oxalate precipitation has been characterised, but the use of organic calcium and phosphate salts appears to mitigate calcium phosphate precipitation. Electron microscopy provides interesting results on NP precipitation, but sample preparation may present technical limitations that affect the interpretation of the results.

## 1. Introduction

Parenteral nutrition (PN) is an essential nutrient delivery route for patients who cannot receive oral or enteral feeding. This method, that consists of intravenous administration of nutrients, encompasses a range of components crucial to maintaining an individual’s nutritional status [1,2]. However, the inherent complexity of parenteral nutritional solutions, combining amino acids, lipids, glucose, mineral salts, vitamins, and other essential elements, poses significant challenges in terms of the stability and compatibility of the components. They are made up of different chemical species that can interact with each other or with external elements, such as storage materials, light, or oxygen [3,4,5]. In addition to this complexity, the calculation of its composition is patient-dependent. The calculation considers their baseline characteristics (anthropometric data, previous nutritional status, etc.) and their clinical evolution (analytical parameters, volume restriction, renal function, and liver function). Due to this personalisation, critical limits may be reached for certain components, which may affect the stability and safety of the admixture [6].

One of the main problems with PN solutions is the formation of precipitates, specifically calcium-phosphate (Ca-P). This problem has received extensive attention in scientific research. The interaction between calcium and phosphorus ions can lead to the precipitation of salts, which compromises the integrity of the PN and puts the patient’s health at risk. In aqueous solutions containing P salts, an equilibrium is established between the three ionic phosphate species: the trivalent phosphate ion and its monobasic (H2PO4-2) and dibasic (HPO4-2) forms. Tribasic phosphate species are not normally present in PNs due to the extreme alkalinity required for ionization. Both mono- and dibasic-P salts can react to form salts with Ca. However, the dibasic salt is the less soluble of the two options. The presence of the latter salt increases the likelihood of Ca-P precipitation at near physiological pH. Because of this, a high pH in the solution increases the probability of precipitation. The pH of PN mixtures is also influenced by other components, such as amino acids or glucose [7,8,9,10,11,12].

In addition to pH, other factors can influence calcium-phosphate precipitation. Increases in temperature or prolonged administration or storage times favour the reactivity of the components. This is due to the increased mobility of the ions in the solution and the long quiescent period of the sample, which allows the formation of precipitates [11]. Macronutrients also influence this process. Ca-P compatibility, for example, increases in the presence of lipids. Cations, such as calcium, interact with the phospholipids that coat the fat globules, decreasing the concentration of free calcium available for precipitation. Amino acids, on the other hand, act as buffers, preventing pH rise and reducing the likelihood of precipitation. In addition, they can form complexes with calcium and phosphorus, reducing the free fraction of these elements in solution. The concentration and nature of the Ca and P salts are another factor to be considered. The higher the concentration, the higher the probability of Ca-P precipitation. On the other hand, there are different types of salts available for Ca and P supply in PNs: organic and inorganic. Due to their dissociation characteristics, the concentrations of free Ca or P are higher when inorganic salts are used. This increases the likelihood of precipitates being generated in solution. Organic Ca salts, such as Ca gluconate (Ca-Glu), are widely used. The use of organic P salts, such as glucose-1 phosphate (G1P) or sodium glycerophosphate (GP), did not become widespread until recently. This was mainly because their use was not registered and approved in several countries. These salts reduce the risk of precipitation, even in the presence of high concentrations of these salts [9,13,14,15,16].

Other studies have shown that certain trace elements can also interact with various nutrients, forming precipitates other than Ca-P precipitates. Certain amino acids in PNs, such as sulphur-containing amino acids (cysteine and methionine) for example, are affected by redox reactions with elements such as zinc, copper, iron, and selenium, giving rise to high-affinity compounds [17]. In addition, cysteine was also found to interact with Ca-Glu to some extent, and to a greater extent than with CaCl_2_. Another example is Ca precipitation with oxalic acid. Oxalic acid is a degradation product of ascorbic acid, a common component of PN [12,18].

The administration of unstable PN, in which precipitating processes occur, can have fatal repercussions for patients. In 1994, an FDA warning report was issued for the occurrence of two cases of death and at least two other cases of respiratory distress. It is believed that these cases may have been due to the infusion of ternary mixtures containing calcium phosphate precipitates [19]. The autopsies of these patients revealed diffuse microvascular pulmonary embolism, with calcium phosphate precipitates found in the lungs. In paediatric patients, calcium and phosphate intake per kg body weight is higher than in the adult population. This, together with the higher temperature of incubators, increases the risk of precipitation. In this regard, the use of in-line filters in PN administration equipment is an effective and safe way to prevent the passage of precipitates or particles >5 µm, such as large lipid droplets [8,20].

Studies in this area focus on Ca-P precipitation. However, there is a high heterogeneity among the studies, both in the salts and macronutrients used and in the analytical methodology [21]. The determination methods vary and are mostly based on different visual inspection techniques with or without aid, such as high-intensity light against black/white backgrounds, turbidimetric analysis or others. Different works have explored new ways to characterize precipitates. The aim is to obtain more data that will allow us to understand the causes of their formation, and thus prevent precipitation. In this sense, electron microscopy is a potentially useful technique for the characterisation of precipitates [22]. This technique can be coupled with atomic absorption spectrophotometry, which provides information on Ca concentration before and after filtering the sample [15,23], and allows us to observe variations in the concentration of the elements to identify the precipitate. Other similar techniques are infrared spectroscopy [7,9] or energy dispersive spectroscopy (EDS) [7,9,15,22,23,24,25]

In this study, we performed a stability analysis of PN solutions by electron microscopy, coupled with EDS. The objective was focused on the determination of precipitates in these solutions.

## 2. Materials and Methods

### 2.1. Sample Preparation

Eight paediatric parenteral nutrition solutions were evaluated in the study (Table 1). The protocol used for their preparation was the PN protocol prescribed for premature infants at the University Hospital Complex of Vigo. The calculation was adjusted to the requirements of a premature newborn weighing 1 kg. In the preparation of these solutions, the rules and procedures relating to the cleaning and disinfection of the area, the use of aseptic techniques, the use of laminar flow cabinets (CFL), and the evaluation of the finished product were followed.

The latest Spanish consensus on the preparation of PN mixtures developed by the Artificial Nutrition Pharmacy Working Group of the Spanish Society of Parenteral and Enteral Nutrition (SENPE) in 2009 [18] establishes both which additives are incorporated and the sequence of addition of the components. According to this protocol, amino acids and glucose will be introduced first into the PN bag. Glucose and lipids will never be mixed directly without amino acids. Monovalent electrolytes (Na and K) shall then be added, followed by phosphate and magnesium. Calcium is added as far as possible from phosphate, thus avoiding localized concentration, which increases the risk of precipitation. The lipid emulsion is incorporated into the mixture of amino acids, glucose, electrolytes, and trace elements, facilitating a visual inspection of the mixture. Finally, the vitamins are added to the samples.

In the first seven samples (PN1–PN7), the nutritional profile was modified according to real clinical practice throughout the first days of life of a newborn. In the last sample (PN8), the changes in composition were exaggerated in the electrolytes to simulate borderline situations in terms of stability.

With this aim in mind, electrolyte concentrations saw a surge of 50–100%, surpassing the maximum amount suggested by the protocol. Macronutrient levels remained at their fundamental baseline. As for lipids, their concentration was elevated, in accordance with the protocol.

A total sample volume of 100mL was prepared for each solution. The amounts and concentrations of all components were progressively varied, according to the centre’s protocol for parenteral nutrition in preterm infants. At day 0, a single stock sample was prepared per solution. These samples were separated into 2 aliquots of 50 mL. One of the aliquots was stored at room temperature (RT) and the other in a refrigerator (4 °C) for 7 days.

This was a coupled study to another study that evaluated lipid emulsion stability on the same samples [26], so the methodology used in terms of storage protocol (time and temperature) was followed.

The original bag was used to store the samples at room temperature (ethylene vinyl acetate (EVA) plastic), without air, and was sealed. Samples stored in the refrigerator were dosed into sterile airless syringes (polypropylene) with a luer-lock cap. For the measurements, the amount needed for each experiment was withdrawn from the syringe or bag. Throughout the process, the samples were shielded from light exposure and maintained in an air-free environment. To prevent microbiological contamination, sterile equipment was consistently employed. Additionally, preparation procedures were conducted in a sterile environment within laminar flow cabinets.

### 2.2. Electron Microscopy

This method was chosen to analyse the occurrence of precipitates in the PN. A scanning electron microscope (JEOL JSM-6700F, Tokyo, Japan) with EDS (energy dispersive X-ray spectroscopy) microanalysis was used. The scanning electron microscope uses electromagnetic lenses to conduct the electron beam to the sample, sweeping its surface and generating different interactions, which allows images with topographic or compositional contrast to be obtained.

To carry out the analysis, after the sample was prepared, it was dosed into enough Eppendorf tubes, separating those to be kept at room temperature and those to be kept in the fridge. On the day of preparation, they were taken to the department where the measurement was to be performed. There, the sample was loaded into a sterile syringe which was attached to a 0.22 µm filter (13 mm PVDF membrane, Durapore^®^, Madrid, Spain), through which 0.5ml of the sample was passed. Then, enough air was passed through the same syringe to filter out the entire liquid. This process was carried out on the same day as the samples were received, i.e., day 0 and day 7. Once all the excess liquid had been removed, the filters were stored for drying and further observation. At day 0, only one filter was stored, with the ambient and refrigerator samples being considered equal. On day 7, the filter corresponding to ambient storage and the other to refrigerator storage were available.

Subsequently, when visualizing, each sample has a pre-preparation consisting of a carbon coating, which is necessary for visualization with the equipment.

The visualization was not carried out on the entire filter. Determinations were made of the precipitates found on the diametral line of the filter, so qualitative data would be available. Photographs were taken, where the size is measured by its longest dimension. The composition of the precipitates was also analysed using coupled EDS microanalysis.

The qualitative nature of the visualisations impeded the evaluation of the storage protocol, so no differences according to the temperature or storage time were evaluated.

## 3. Results

Precipitates were visualized in all samples, even at day 0. In some samples, these precipitates were >25 µm in size. The precipitates were classified according to the visual appearance of their structure as crystalline or amorphous structures.

### 3.1. Crystalline precipitates

**Figure 1:**
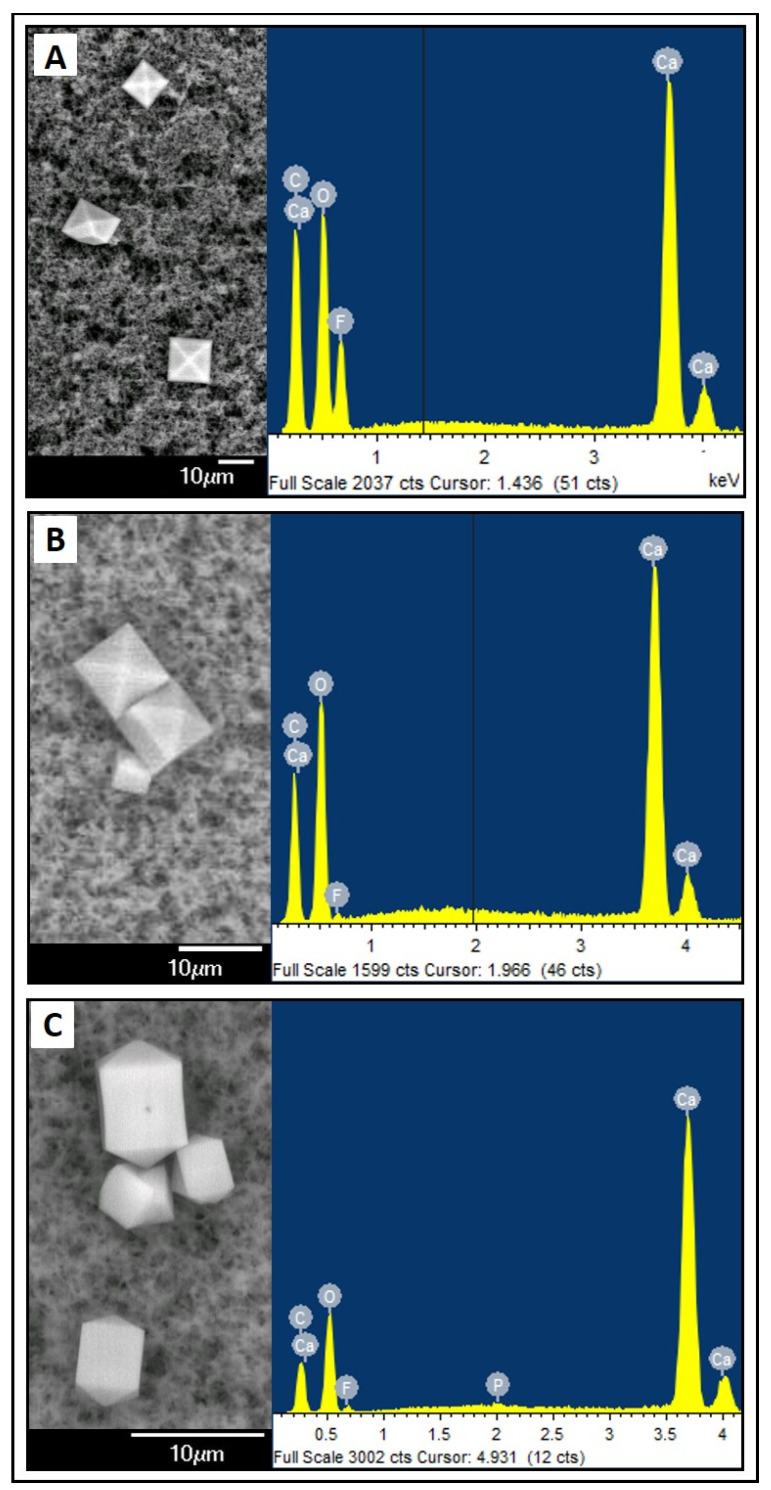
Examples of crystalline precipitates with a calcium-based composition. Electron microscopy photograph and EDS compositional analysis. (**A**) sample PN1A7; (**B**) sample PN2A7; (**C**) sample PN4A7.

**Figure 2:**
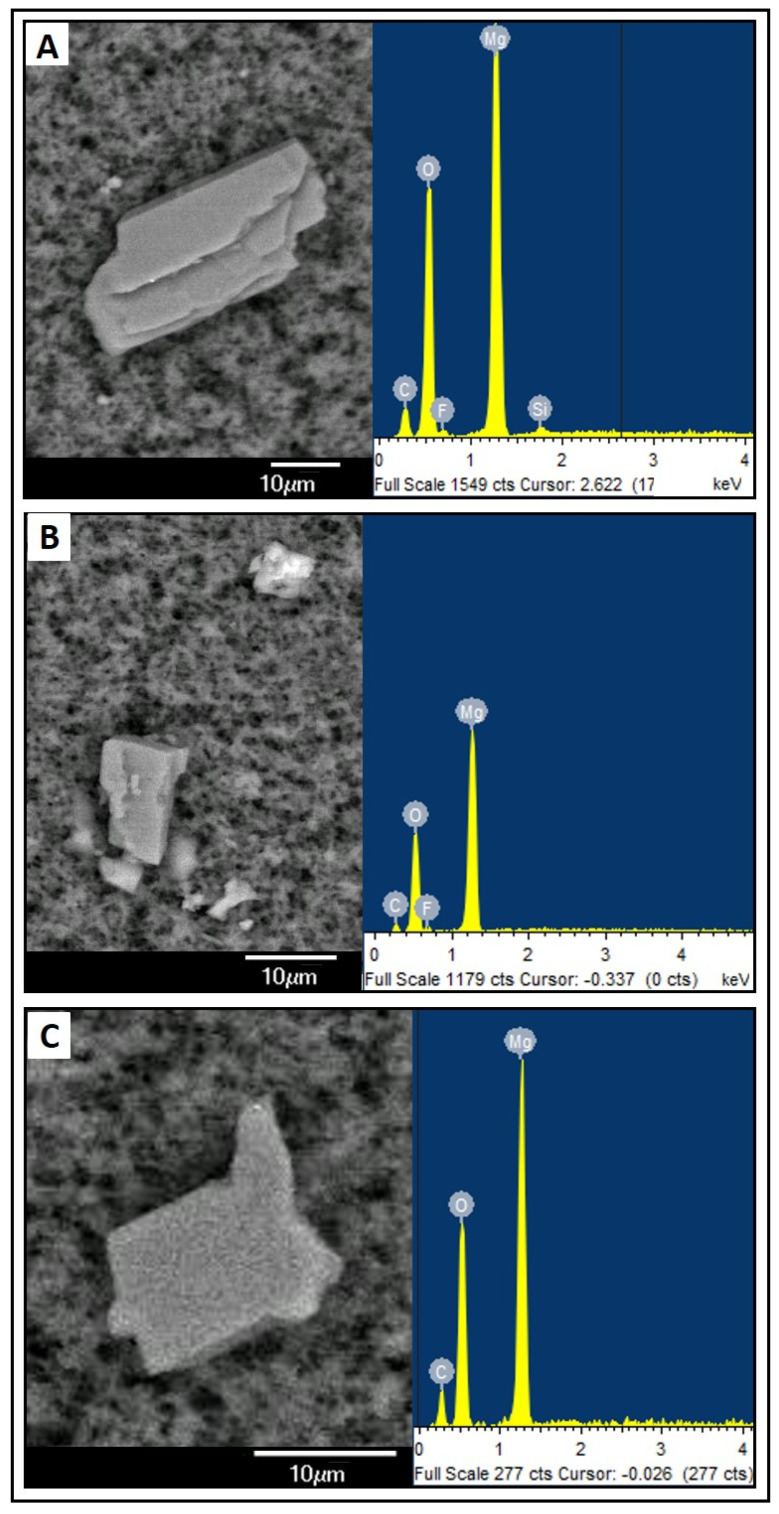
Examples of crystalline precipitates with a magnesium-based composition. Electron microscopy photograph and EDS compositional analysis. (**A**) sample PN5A7; (**B**) sample PN5N7; (**C**) sample PN6A7.

Crystalline precipitates composed of calcium, carbon, and oxygen (Ca-C-O) (Figure 1) or magnesium, carbon, and oxygen (Mg-C-O) (Figure 2) were observed. These were the most repeated compositions in all reports. In addition, these crystalline precipitates composed of calcium were sometimes associated with chlorine or phosphorus, although their occurrence was minor. In the magnesium-based composite precipitates, silicon was also detected with some frequency. Fluorine is observed in the EDS plots, but this element is contamination coming from the filter composition (PVDF).

In terms of shape, more diverse structures were observed. One of the most common ones corresponds to a rectangular prism shape with a pyramidal base in calcium-based precipitates. Similarly, other structures were found that had calcium in their composition in different shapes. Structures with a “laminar” shape were also observed, often associated with the Mg-C-O composition.

### 3.2. Amorphous Precipitates

**Figure 3:**
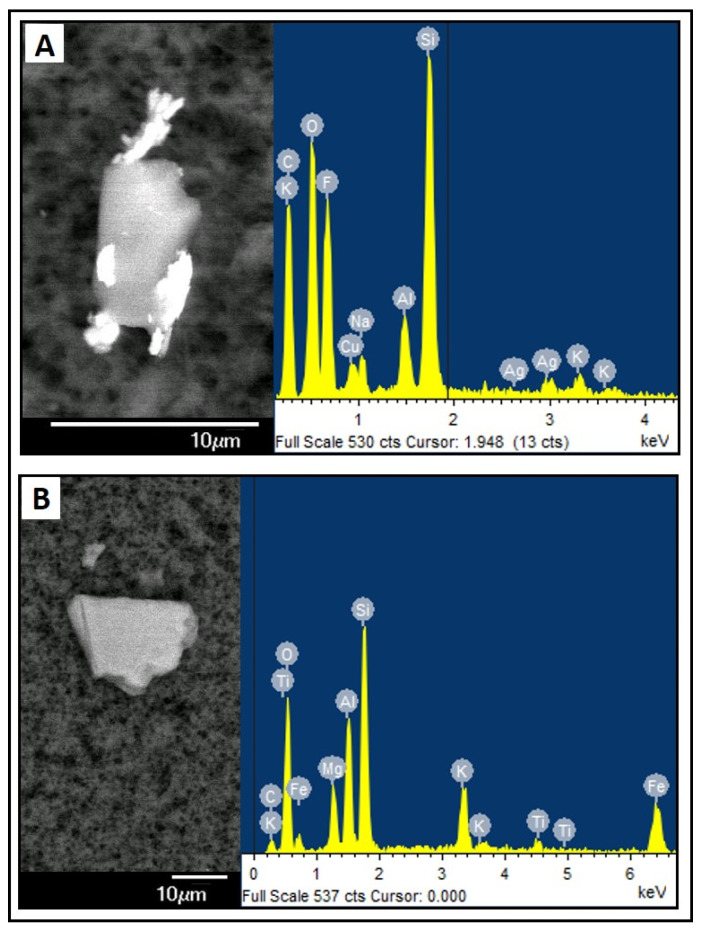
Examples of amorphous structures with varied composition. Electron microscopy photograph and EDS compositional analysis. (**A**) sample PN2A0; (**B**) sample PN8N7.

These structures had very heterogeneous compositions, including elements not present in the initial composition of our samples: Fe, Cu, Ag, Ti, Si, Al, Al, Mo, Ta, Sn, Cr, I, Ni, Mn (Figure 3). Our samples were prepared with OligoCinc Fresenius Kabi^®^ (Barcelona, Spain), which explains the appearance of Zn, but not of the other elements (Section 2).

## 4. Discussion

Our study explored the use of electron microscopy, coupled with EDS, for the identification and characterisation of precipitates in PN samples for preterm infants. With this technique, precipitates of different shapes, sizes, and compositions were identified. These characterisations were performed with a descriptive aim and the analyses were qualitative.

On this basis, the first thing to note is the considerable presence of precipitates. They were observed in all the samples visualised. Sometimes these precipitates were large, which is potentially dangerous for patients. This was not an expected result, at least in the first samples (PN1–PN7) as their composition was that commonly used in clinical practice. On the other hand, due to the composition of PN8, the occurrence of precipitates would be reasonable. In this sample, the electrolyte concentrations were exaggerated, and the amino acid concentration was reduced in order to force the state of instability.

In our study, we have obtained a large number of reports with precipitates of a crystalline form, with a Ca-C-O composition and a prism shape with a pyramidal base. These observations correlate with calcium oxalate precipitates. It is described in the literature that ascorbic acid is the least stable vitamin and its degradation leads to an end product, oxalic acid, which can react with the calcium ions present in solution. This degradation is mediated by oxygen that may come into contact with the PN at the time of preparation [12]. In the clinical setting, where the air in the bag is released at the time of preparation when the bag is closed, it is unlikely that the remaining oxygen could cause degradation. In our samples, the handling of the microscopic analysis resulted in increased O_2_ contact with the sample.

This characterisation of calcium oxalate in our samples was also confirmed by other photographs published in similar studies. Our results are consistent with those described by Lázaro Cebas A et al. [27]. In this article, the precipitation limits were studied in paediatric PNs using organic sources of calcium and phosphorus also using electron microscopy associated with EDS. Filter preparation was performed on the total prepared sample (100 mL). As in our study, air was injected through the filter to remove the amount of liquid and dried for 24 h. They observed a large amount of precipitates and amorphous particles on most of the filters. These precipitates varied in size between 5–15 µm, and were likely to have originated due to crystallisation processes during the storage of the filters. The amorphous particles were compatible with residues from the preparation process (gums, crystals, fibres, etc.). Ambados et al. [28] analysed the incompatibility of trace elements during the preparation of PN solutions. They also observed crystals that could be caused by the interaction between calcium and oxalic acid.

As for the analysis of the structures found with a magnesium-based compositional pattern, their characterisation was more difficult because there are no published reports on parenteral nutrition samples with precipitates of this type. From its composition, it could correspond to magnesium oxide or magnesium carbonate crystallisation. Additionally, since silicon is also commonly detected, it could correspond to magnesium silicates.

Calcium oxalate crystals were the most characteristic precipitates found in our samples. The characterisation of other specific precipitates is more complicated. The occurrence of other calcium-based precipitates, such as calcium chloride, would be possible, as compositional spectra with calcium and chlorine are obtained in some samples. Compositions with calcium and phosphorus are also found, which would imply the formation of calcium phosphate, but these reports were less common. This observation is consistent with the previously mentioned study, similar to ours, by Lázaro Cebas et al. [27], where most of the precipitates found were rectangular in shape and phosphorus was not detected in their composition. This suggests that organic phosphorus salts (glycerophosphate in our case) greatly hinder the precipitation processes between calcium and phosphorus.

Calcium phosphate precipitates form when there is an oversaturation of calcium and phosphate ions in the PN solution. In this situation, the ions may start to bind together to form calcium phosphate crystals. This is the most studied precipitate in PN samples. The concern was the greatest when inorganic salts were used. With the introduction of the organic salts of phosphorus (sodium glycerophosphate and glucose-1-phosphate) and calcium (calcium gluconate), calcium-phosphate compatibility increases and calcium phosphate precipitation is less probable [16].

However, we must not lose sight of this point, as the formation of precipitates is a crucial point in terms of the safety of the therapy. The fact that the risk is reduced by the use of organic salts does not mean that precipitation cannot occur. The characterisation of precipitates in PN mixtures is described in the literature even when using organic Ca and P salts. Mark MacKay et al. [13] observed that precipitation could occur at high concentrations of organic calcium and phosphorus salts, although all samples remained within the limits set by the USP. In the study by Christos Athanasiou et al. [29], the early formation of precipitates was observed using the organic salts of Ca and P. It was deduced that Ca is the most important factor, as no precipitation was observed in the batches of samples without Ca compared to the rest. Chaieb et al. [23] found precipitates in PNs with organic salts. This was more pronounced in samples with higher Ca and P concentration, no lipids, and a lower amino acid concentration. Along these lines, in the study by Mansi J. Parikh et al. [9], where organic Ca salts were used but inorganic P salts were used, dibasic calcium phosphate was characterised by infrared spectroscopy. They suggest an increased likelihood of precipitate formation at low amino acid concentrations and high pH values.

Finally, the study by Joy et al. [7] examined the compatibility between calcium and phosphate in low osmolarity PN mixtures intended for peripheral administration. In two of the samples studied, with the highest calcium and phosphorus concentrations, precipitation was observed simply by visual inspection. Precipitates were confirmed using polarised light microscopy with X-ray diffraction and infrared spectroscopy. It was characterised as Brushite or calcium hydrogen phosphate dihydrate (dibasic calcium phosphate).

On the other hand, our study has also analysed the occurrence of unusual elements in the PN samples. In the analysis carried out using EDS microanalysis, the presence of Fe, Zn, Cu, Ag, Ti, Si, Al, Mo, Mo, Ta, Sn, Cr, I, Ni, Mn was detected. The only element intentionally added in the preparation of the samples was Zn (Meinsol Oligo-zinc Fresenius Kabi^®^ Barcelona, Spain). The remaining elements could be associated with the presence of impurities or contaminants introduced during the preparation protocol (vials, broken glass or plastic ampoules, sterile gauze fibres, rubber remains from perforated caps, metal needles, etc.). Ball et al. [30] studied the contamination in PN solutions by small solid particles, such as fibres, dust, or plastic fragments. These particles can cause serious complications, such as thrombosis, pulmonary embolism, or renal failure. In addition, certain industrially manufactured products used in the preparation of PNs are known to contain traces of undesirable elements, e.g., aluminium [31]. In organic salts (calcium and phosphate) and glucose solutions, a higher presence of this element is observed [32,33]. This aluminium usually derives from the raw material itself or from the manufacturing and packaging processes.

Intravenously administered aluminium is deposited in the bones, liver, brain, and other tissues. Newborns are a particularly susceptible population to aluminium toxicity due to their renal and hepatic immaturity. This group of patients also receives the highest amount of aluminium per kilogram of body weight with PN, as they have higher Ca and P requirements [34,35].

As mentioned above, the compatibility of Ca-Glu with organic P salts is much more favourable than the inorganic salts of both ions, however, their aluminium content is higher. Although most guidelines suggest the use of organic salts in order to improve compatibility and safety by decreasing the possibility of precipitation of calcium-phosphate salts, some studies explore the possibility of using organic P salts with inorganic Ca salts, such as CaCl_2_, in order to decrease the total amount of aluminium present in the PN without compromising the formation of precipitates [13,14,36].

Finally, one of the main limitations of this study was the preparation of the filter for microscope visualisation. A poor extraction of the sample after filtration could lead to the crystallisation of the precipitates by the evaporation of the remaining sample. Therefore, it is likely that the observed precipitates were formed after the filtration of the sample, during the storage of the filters prior to visualisation (Section 2). Therefore, it could not be claimed that everything found during visualisation was present in the PN sample. Our results suggest that such precipitates could form in PNs with similar compositions, but with concentrations close to the saturation limits. This situation is not expected in routine clinical practice. Additionally, the small number of samples tested is another limitation present in this study, which prevents drawing conclusions with direct clinical applications. However, our results improve the understanding of potential precipitation processes in PN mixtures. Our data are relevant as a starting point for future larger studies. Furthermore, it positions electron microscopy as a very useful technique for stability studies in parenteral nutrition. It seems likely that image analysis software could be coupled to electron microscopy to extend the scope of similar studies with quantitative measurements.

## 5. Conclusions

Different types of precipitates can form in PN samples, usually calcium- or magnesium-based compounds, although they are not expected to form under the conditions of routine clinical practice.

Calcium oxalate precipitation has been characterised, but the use of organic calcium and phosphate salts complicates calcium phosphate precipitation.

For the identification of precipitates formed in PN samples, electron microscopy provides interesting results, but sample preparation may present technical limitations that affect the interpretation of the results. Crystallisation processes could be mistaken as precipitation processes.

Electron microscopy reveals the presence of elements that are not intentionally added in the PN, possibly impurities from commercial components used in its preparation, or incorporated during the analytical process.

## Figures and Tables

**Table 1:** Details of the composition of the paediatric samples used in the study.

Sample	N (g/L)	Prot (g/L)	Gluc (g/L)	Lip (g/L)	Na (mMol/L)	K (mMol/L)	Mg (mMol/L)	Ca (mMol/L)	P (mMol/L)	OSM (mOsm/L)	CAN (mMol/L)
PN1	3.14	19.64	71.43	7.14	20.00	10.00	1.25	10.00	10.00	743.33	750
PN2	3.38	21.10	80.25	11.38	30.00	15.00	2.25	15.00	15.00	878.62	1149
PN3	3.56	22.22	87.11	14.78	35.00	20.00	2.60	17.50	17.50	969.77	1341
PN4	3.70	23.13	92.60	17.50	40.00	30.00	3.00	20.00	20.00	1054.23	1542
PN5	4.20	26.25	106.80	21.60	40.00	30.00	3.00	20.00	20.00	1178.95	1542
PN6	4.70	29.38	121.00	25.80	50.00	35.00	3.50	22.50	25.00	1350.08	1749
PN7	5.20	32.50	135.20	29.90	60.00	40.00	4.00	25.00	30.00	1520.80	1956
PN8	3.14	19.64	71.43	40.00	80.00	50.00	5.00	30.00	40.00	1150.91	2370

PN1–PN8: Paediatric samples; N: nitrogen; Prot: protein (estimated using conversion factor 6.25 g Protein/g nitrogen; commercial source used Aminoven Infant 10% Fresenius Kabi^®^ Barcelona, Spain); Gluc: glucose (Glucose 50% Grifols^®^ Barcelona, Spain); Lip: lipids (Lipoplus 20% Braun^®^ Melsungen, Germany); OSM: osmolarity; CAN: critical aggregation number, calculated according to the cation concentration to analyse its relationship to stability (CAN = a + 64 b + 729 c; where a, b and c are the sum of the concentrations (mmol/L) of mono-, di- and trivalent cations, respectively). Other components used: Sodium chloride 20% Braun^®^, Potassium acetate 1 M Braun^®^, Sodium glycerophosphate: Glycophos Fresenius Kabi^®^, Calcium gluconate: Suplecal Braun^®^, Magnesium sulfate 15% Genfarma^®^ Madrid, Spain, Vitamins: Vitalipid Fresenius Kabi^®^, Trace elements: Meinsol Oligo-zinc Fresenius Kabi^®^, Water for injection Grifols^®^.

## Data Availability

Restrictions apply to the datasets. The datasets presented in this article are not readily available because large data set. Requests to access the datasets should be directed to correspondence authors.
